# The *Bacillus subtilis* TRAP Protein Can Induce Transcription Termination in the Leader Region of the Tryptophan Biosynthetic (*trp*) Operon Independent of the *trp* Attenuator RNA

**DOI:** 10.1371/journal.pone.0088097

**Published:** 2014-02-04

**Authors:** Natalie M. McAdams, Paul Gollnick

**Affiliations:** Department of Biological Sciences, University at Buffalo, Buffalo, New York, United States of America; University of Groningen, Groningen Institute for Biomolecular Sciences and Biotechnology, Netherlands

## Abstract

In *Bacillus subtilis*, transcription of the tryptophan biosynthetic operon is regulated by an attenuation mechanism. When intracellular tryptophan levels are high, the TRAP protein binds to the 5′ leader region of the nascent *trp* mRNA and induces transcription termination prior to the structural genes. In limiting tryptophan, TRAP does not bind and the operon is transcribed. Two competing RNA secondary structures termed the antiterminator and terminator (attenuator) can form in the leader region RNA. In prior attenuation models, the only role of TRAP binding was to alter the RNA secondary structure to allow formation of the attenuator, which has been thought function as an intrinsic transcription terminator. However, recent studies have shown that the attenuator is not an effective intrinsic terminator. From these studies it was not clear whether TRAP functions independently or requires the presence of the attenuator RNA structure. Hence we have further examined the role of the attenuator RNA in TRAP-mediated transcription termination. TRAP was found to cause efficient transcription termination in the *trp* leader region *in vivo* when the attenuator was mutated or deleted. However, TRAP failed to induce transcription termination at these mutant attenuators in a minimal *in vitro* transcription system with *B. subtilis* RNA polymerase. Further studies using this system showed that NusA as well as the timing of TRAP binding to RNA play a role in the observed differences *in vivo* and *in vitro*.

## Introduction

**Figure 1:**
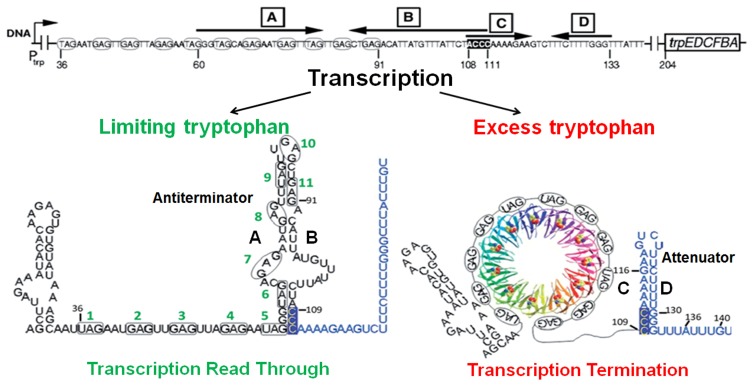
Model of transcription attenuation of the *B. subtilis trp* operon. Bold black letters designate the complementary strands of the attenuator (C/D) (highlighted in blue) and antiterminator (A/B) RNA structures. TRAP is shown in a ribbon diagram with each subunit in a different color. The 11 (G/U)AG repeats of the TRAP binding site are circled and numbered in green. Small black numbers indicate RNA residues relative to the start of transcription. When tryptophan is limiting, the AB antiterminator RNA structure forms, allowing read through of the *trp* operon. In excess tryptophan, TRAP binds to the nascent RNA and prevents formation of the antiterminator structure, which allows formation of the attenuator, leading to transcription termination.

Expression of the *trpEDCFBA* (*trp*) operon, which contains six of the seven genes required for tryptophan biosynthesis in *B. subtilis*, is regulated by the *trp* RNA-binding attenuation protein (TRAP) [1], [2]. Transcription of this operon is regulated by an attenuation mechanism. The model for this mechanism is shown in Figure 1. Two mutually exclusive RNA secondary structures called the antiterminator and the terminator (attenuator), which form in the 203 nt 5′ leader region of the *trp* transcript upstream of *trpE*, are proposed to control transcription of the operon [3], [4]. When the intracellular concentration of tryptophan is in excess, TRAP is activated to bind to a target in the leader region of *trp* mRNAs. This binding prevents formation of the antiterminator structure, which allows formation of the attenuator and the *trp* genes are not expressed. Conversely, when tryptophan is limiting, TRAP does not bind RNA, and the antiterminator structure forms, which allows transcription of the *trp* genes (Figure 1) [5].

TRAP is composed of 11 identical subunits, each encoded by the *mtrB* gene [6]. These 11 subunits, each composed of 75 amino acids, assemble into a symmetric ring complex [7]. TRAP is activated to bind RNA by binding 11 tryptophan molecules in hydrophobic pockets between adjacent subunits [8]. The TRAP binding site in the *trp* mRNA leader region is composed of 11 repeats of GAG or UAG separated by 2 or 3 nonconserved spacer nucleotides [9]. When bound to TRAP, the RNA wraps around the outside of the protein ring [7], [9].

In the original model for TRAP-mediated control of transcription of the *trp* operon, the only role of TRAP was to alter the secondary structure of the leader RNA (Figure 1) [10]. TRAP binding to the RNA prevents the antiterminator structure from forming, thereby allowing the competing attenuator to form, which was proposed to act as an intrinsic terminator to cause termination [3], [10]. However, several lines of evidence indicate that TRAP plays an additional more direct role in causing transcription termination. The first indication of this came in 1995 from *in vivo* studies of *trpE′-′lacZ* fusions by Merino *et al.* [11]. They found that deleting the terminator stem-loop (from +108 to +133: see Figure 1) reduced but did not eliminate TRAP-mediated regulation of transcription in the leader region. At that time there was no clear explanation for this observation. More recently we have shown that the terminator segment in the *trp* leader region does not function as an intrinsic terminator, and that efficient termination only occurs in the presence of TRAP bound to the nascent *trp* transcript [12]. Together these observations suggest that TRAP plays an additional role in the attenuation mechanism beyond altering the RNA secondary structure.

Canonical intrinsic terminators are composed of a GC-rich stem-loop followed by 7–9 U residues [13]. The base-paired C/D stem of the terminator in the *trp* leader region contains 6 A:U and only 4 G:C pairs (Figure 1). Moreover, the U-stretch following the stem is interrupted twice at A136 and G140 (Figure 1). We have shown that the combination of these features results in this structure being a weak intrinsic terminator [12]. Nevertheless efficient termination does occur in the presence of TRAP bound to the nascent *trp* transcript. Thus the *trp* leader terminator is unusual among bacterial terminators in that it is neither Rho-dependent nor fully intrinsic [12]. Hence, in this paper we will refer to the segment of the *trp* leader region from +109 to +140 (Figure 1) as the *trp* attenuator rather than as a terminator.

From our prior studies [12] it was not clear whether the presence of the attenuator RNA is required for TRAP-mediated termination. Hence, we have examined the role of the *trp* leader RNA in TRAP-mediated transcription termination. To do so, changes were made in the hairpin stem and/or in the U-stretch of the attenuator and the ability of TRAP to induce transcription termination at these mutant attenuators was assessed both *in vivo* and *in vitro*. *In vivo*, TRAP was able to induce termination within each of the mutant *trp* leader regions tested; even those lacking the attenuator hairpin stem and/or U-stretch. Moreover, termination occurs at approximately the same location, relative to the start of transcription, in both the WT and mutant leader regions. The only requirement for TRAP-mediated termination is an A+U-rich segment downstream of the TRAP binding site. Hence, it appears that *in vivo* the attenuator plays a minor role, if any, in TRAP-mediated termination. In contrast, even minor changes to the attenuator eliminated TRAP-mediated termination when examined using a simple *in vitro* transcription system with *B. subtilis* RNAP. These observations suggest that additional factors are absent in this minimal *in vitro* system as compared to *in vivo*. Further analysis showed that NusA and the timing of TRAP binding to RNA contribute the observed differences *in vivo* and *in vitro*.

## Material and Methods

### Bacterial strains and transformations

*E. coli* K802 was used as a host for plasmid construction and propagation. *B. subtilis* BG2087 (*argC4*) and BG4233 (*argC4* Δ*mtrB*) were used as hosts for transformation and integration of gene fusions. BG4233 contains a deletion in *mtrB* (from codons 7–62), which encodes TRAP [14]. *B. subtilis* was transformed by natural competence [15], and colonies were selected on plates containing Vogel and Bonner (VB) minimal salts [16], 0.2% acid-hydrolyzed casein, 0.2% (w/v) glucose, 50 µg/ml 5-bromo-4-chloro-3-indolyl-βD-galactopyranoside (X-gal), 10 µg/ml L-arginine, and 5 µg/ml chloramphenicol.

### Protein purification

TRAP was expressed in *E. coli* BL21(DE3) (Novagen, Madison, WI) and was purified as described previously [17]. *B. subtilis* NusA was expressed in *E. coli* strain M15(pREP4) using the plasmid pQE30-NusA, which was a gift from Tina Henkin, encoding a N-terminal His-tagged *B. subtilis* NusA protein and was purified using Qiagen Ni-NTA beads as per manufacturer's instructions.

### Plasmids and gene fusions

Mutations in the *trp* attenuator were created using QuikChange Site-Directed Mutagenesis (Stratagene) using pUC*trpL* plasmid as the template [12]. pUC*trpL* contains a 730-bp EcoRI-HindIII fragment including the *B. subtilis trp* promoter and leader sequence and the start of *trpE* (−411 to +318 relative to the start of transcription). The resulting plasmids with mutant leader sequence were used as templates for PCR to generate transcriptional fusions with *lacZ*. PCR was used to introduce *EcoRI* and *BamHI* restriction endonuclease sites at the ends of a DNA fragment containing the *trp* promoter, leader region, and the first 45 codons of *trpE* followed by a stop codon. Transcriptional fusions with *lacZ* were made by digesting the PCR product with *EcoRI* and *BamHI*, and ligating the DNA into similarly digested pDH32 [18]. DNA sequencing was used to confirm successful mutagenesis and subcloning. The resulting plasmids were linearized with *PstI*, transformed into *B. subtilis* BG2087 and BG4233, and integrated into the *amyE* locus by homologous recombination, and the *amyE^−^* phenotype was confirmed on starch plates [18].

### β-galactosidase assay

*B. subtilis* strains containing *lacZ* reporter fusions were grown in 0.2% acid-hydrolyzed casein, 1X MOPS solution pH 7 (40 mM MOPS buffer, 50 mM KCl, 0.5 mM MgSO_4_, 4 mM Tricine, and 10 mM NH_4_Cl), 1.3 mM K_2_HPO_4_, 10 µM FeCl_3_, 5 µg/ml arginine, and 5 µg/ml chloramphenicol in the presence or absence of 50 µg/ml L-tryptophan at 37°C to an absorbance between 0.4 and 0.6 at 600 nm. Cells (1.5 ml) were pelleted, washed with TE buffer pH 8, and resuspended in 1.5 ml of Z-buffer (60 mM Na_2_HPO_4_, 40 mM NaH_2_PO_4_, 10 mM KCl, 1 mM MgSO_4_, and 50 mM β-mercaptoethanol). Cells were lysed by addition of lysozyme to 0.1 mg/ml with incubation at 37°C for 5 minutes, followed by addition of Triton X-100 to a final concentration of 0.1%. Cell lysate (0.1 ml) was added to 0.9 ml of Z-buffer and assayed for β-galactosidase activity [19]. Each value reported is the average of two or three independent experiments, each performed in triplicate.

### RNase protection assay (RPA)

*B. subtilis* cells were grown in LB overnight at 37°C and then diluted 1/20 into 100 ml of LB. The cells were grown at 37°C to an A600 of 0.6, harvested, and resuspended in a hot phenol mixture containing equal volumes of RNA extraction buffer (0.5 M NaCl, 10 mM EDTA, 125 mM Tris-Cl pH 7.5, and 0.2% SDS), chloroform, and phenol. The mixture was incubated at 75°C for 10 minutes with shaking and the aqueous phase was collected following centrifugation. RNA was then precipitated by adding 3 volumes of ethanol, followed by incubation at −20°C for 1 hour [3].

PCR was used to generate a DNA template with a T7 promoter positioned to transcribe antisense RNA to the *trp* leader region. RNA made from this template is complementary to +1 to +376 of the *trp* leader region, and contains 15 bases of non-complementary sequence at each end. The DNA templates were transcribed *in vitro* using T7 RNAP as described previously [20]. For RNase protection assays, 25 µg of cellular RNA was incubated with 10,000 cpm (∼50–60 fmol) of ^32^P-labeled antisense probe in hybridization buffer, and assays were performed as per manufacturer's instructions (RPA III™ Ambion). Reaction products were separated on 8% denaturing polyacrylamide gels, dried, and exposed to phosphorstorage screens. Size markers were created by T7 RNAP transcription of two DNA templates containing substitutions in the U-stretch of the *trp* attenuator. PCR was used to place a T7 promoter upstream of the template with an XbaI site from +133–138, or a PstI site from +134–139 relative to the start of transcription. The 137 nt marker was generated by transcription of the XbaI cleaved template. Similarly transcription of the No Binding Site template cut with PstI yielded a 134 nt RNA marker [12].

### *In vitro* transcription attenuation assay

DNA templates for *in vitro* transcription attenuation assays were created by PCR amplification of pUC*trpL* plasmids using the M13 Forward and M13 Reverse primers. PCR products were purified on 1% agarose gels using the QIAGEN MinElute Gel Extraction Kit. Transcription reactions contained 50 µg/ml *B. subtilis* σ^A^ RNA polymerase (RNAP), 20 nM DNA template, 20 mM Tris-HCl (pH 8.0), 4 mM MgCl_2_, 0.1 mM EDTA, 4 mM spermidine, 5 mM DTT, 1 mM tryptophan, 500 µM NTPs (unless otherwise noted in the figures), 1 µCi [α-^32^P] UTP 3000 Ci/mmol [12], and 0–0.5 µM TRAP. Reactions were incubated at 37°C for 15 minutes, and then stopped by adding an equal volume of stop solution (95% formamide, 20 mM EDTA, 0.02% bromophenol blue, and 0.02% xylene cyanol). The samples were heated at 95°C for 2 minutes, and the resulting RNAs were separated on 6% denaturing polyacrylamide gels. The gels were dried, exposed to a phosphorstorage screen, and quantified using IMAGEQUANT software (GE Healthcare). The number of U residues in the terminated and read through transcripts were used when calculating the molar percentage of termination [12].

For block-and-release transcription assays, a cleavage-defective mutant E111Q EcoRI (EcoRI*) protein was added to the DNA template prior to initiating the transcription reaction. EcoRI* bound to the DNA blocks the transcription elongation complex (TEC) such that the active site of RNAP is stalled 12–13 nts upstream of the first G of the EcoRI site [21]. EcoRI* can be dissociated from the DNA by addition of 0.5 M KCl to allow transcription to resume [12]. EcoRI* was purified as described previously [12]. QuikChange (Stratagene) site-directed mutagenesis was used to introduce an EcoRI recognition site (GAATTC) starting at +116 (relative to the start of transcription) of the *trp* leader DNA in pUC*trpL* to generate the plasmid pUC*trpL*Eco116. Since the Mismatch Mutant alters the ability to place the EcoRI restriction site at +116, an alternative analogous C125G Mismatch mutation was examined in this assay.

The *trp* leader region was previously found to contain a cryptic promoter that allows transcription to initiate at +37 *in vitro* [22]. We used PCR amplification with the pUC*trpL*Eco116 plasmid as template and the M13 reverse primer together with a +37*trpL* primer (5′ CAGCTTGACAAATACACAAGAGTGTGTTATAATGCAATTAGAATG 3′) that binds from +31 to +42 in the *trp* leader region and converts the cryptic promoter into a consensus promoter (−35 TTGACA and −10 TATAAT separated by 17 bases). This +37*trpL* template directs transcription to initiate at +37, and promotes more efficient transcription initiation at low NTPs [22]. Prior to initiating transcription, EcoRI* (130 nM) was allowed to bind to the DNA template (20 nM) for 5 min at 37°C. Single round *in vitro* transcription reactions were initiated in the absence of CTP with 50 µg/ml *B. subtilis* σ^A^ RNA polymerase (RNAP), 20 mM DNA template, 20 mM Tris-HCl (pH 8.0), 4 mM MgCl_2_, 0.1 mM EDTA, 4 mM spermidine, 5 mM DTT, 8 µM ATP and GTP, 2 µM UTP, and 1 µCi [α-^32^P] UTP 3000 Ci/mmol, and were incubated at 37°C for 10 minutes [12]. Transcription of this template in the absence of CTP allows initiation and elongation from +37 to +65. TRAP (0.5 µM) was then added, and transcription elongation up to the EcoRI* block at +116 was induced by addition of all 4 NTPs at 30 µM in the presence of 0.1 mg/ml heparin, and 1 mM tryptophan at 37°C for 10 minutes. The EcoRI* block was dissociated from the DNA by addition of KCl to a final concentration of 0.5 M, and transcription was continued at 37°C for 10 minutes. Reactions were stopped and RNA visualized as stated above.

## Results

### TRAP induces efficient transcription termination within the *trp* leader region *in vivo* when the attenuator is mutated

**Figure 2:**
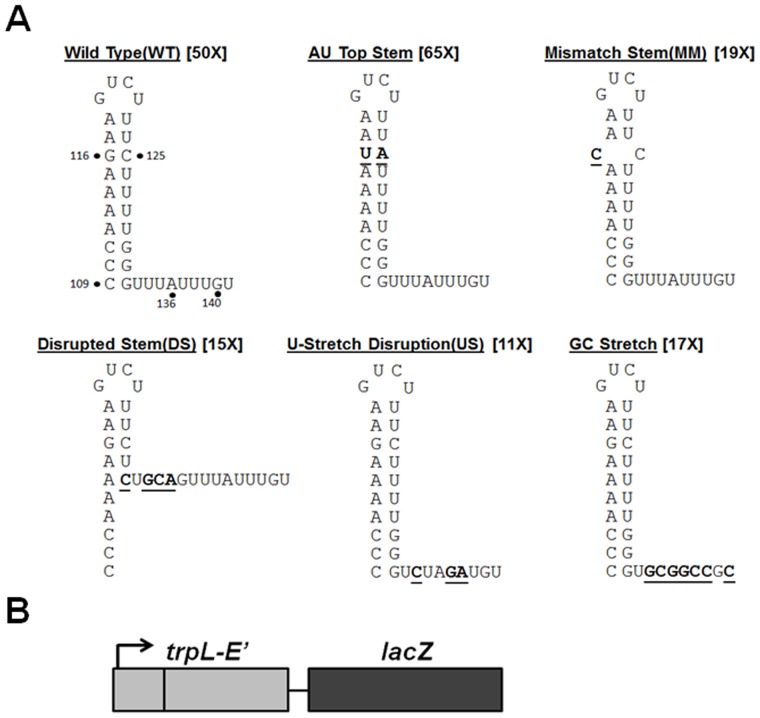
Substitutions in the stem-loop and U-stretch of the *trp* attenuator. **(A)** Diagram depicting the WT and mutant *trp* attenuators with altered bases in the stem-loop structure or U-stretch. Substitutions are indicated in bold and are underlined. Numbers adjacent to structure names indicate fold TRAP regulation observed *in vivo* using β-galactosidase assays (results in Table 1). **(B)** Schematic representation of the transcriptional reporter fusions with *lacZ* that were used to examine TRAP-mediated regulation of transcription termination *in vivo*. The arrow represents the *trp* promoter and *trpL* refers to the regulatory leader region (See Methods for details about each gene fusion). Each *trpE′-′lacZ* fusion was integrated into the *B. subtilis* genome at the *amyE* locus.

The *B. subtilis trp* attenuator is unusual among characterized bacterial transcription terminators. While it contains features in common with intrinsic terminators, including a stem-loop structure followed by a U-stretch (Figure 2A), efficient transcription termination at the *trp* attenuator only occurs when TRAP is bound to the nascent *trp* transcript [12]. Here we have further examined the role of the attenuator in TRAP-mediated regulation of the *trp* operon. To determine which *cis*-acting elements are required for TRAP-mediated termination, mutant *trp* leader templates were generated that contain changes in the stem-loop and/or in the U-rich tract. None of these changes altered the 11 (G/U)AG TRAP binding site (Figure 2A). We examined TRAP-mediated transcription termination at these altered attenuators both *in vivo* and *in vitro*.

The base-paired stem of the *trp* attenuator has a low (40%) GC content as compared to canonical intrinsic terminators [13]. Moreover, it has been shown that this low GC content is in part responsible for the weak intrinsic termination activity of the attenuator [12]. We further examined the role of this stem-loop structure in TRAP-mediated termination by making three sets of changes within the stem, and assessed the effects of these changes on the ability of TRAP to induce termination. The first change replaced the only GC pair (G116/C125) near the top of the stem with an AU pair (Figure 2A AU Top Stem). The second change created a mismatch at this position (Figure 2A Mismatch Stem). Finally, we made substitutions that disrupted six base pairs at the base of the stem, which likely eliminates formation of the stem entirely (Figure 2A Disrupted Stem).

TRAP-mediated termination at each of these mutant attenuators was assessed *in vivo* using transcriptional *trp* operon *lacZ* fusions. Each fusion contains the *trp* promoter and regulatory leader region (either WT or mutant), the first 40 codons of *trpE* and then a stop codon, followed by the entire coding segment of *lacZ* (Figure 2B). Our transcriptional fusions were designed this way so as to allow direct comparison with prior *in* vivo studies [10], [12], [23], all of which used similarly designed operon fusions. Each fusion was integrated as a single copy into the *amyE* locus of the genomes of two different *B. subtilis* strains: BG2087, which contains a WT gene for TRAP, and BG4233 in which the gene for TRAP is deleted. Each strain was grown in the absence and presence of exogenous tryptophan. As expected none of the fusions showed significant regulation in response to tryptophan in BG433, which lacks TRAP. The level of β-galactosidase produced from all the fusions in BG4233 was greater than those seen for the same fusion in BG2087 grown in the absence of tryptophan. This difference reflects endogenously produced tryptophan in BG2087, which is *trp*+. Hence to assess the full ability of TRAP to regulate transcription at each attenuator, β-galactosidase production in the absence of TRAP (BG4233) was compared to that in the strain containing TRAP grown in the presence of tryptophan.

The fusion containing the WT *trp* attenuator was regulated approximately 18-fold in response to exogenous tryptophan in BG2087 (Table 1: 106U −Trp vs 6U +Trp), and overall approximately 50-fold by TRAP (BG4233: 286U vs BG2087 +Trp: 6U). Prior studies measuring changes in mRNA levels from a similar *trpE′-′lacZ* fusion in the same *B. subtilis* strains estimated that TRAP mediated approximately 80-fold regulation of transcription [11].

**Table 1:** **Effects of mutations in the** ***trp*** **attenuator on TRAP-mediated transcription termination** ***in vivo***.

		β-Galactosidase activity (U)^b^			
Regulatory Region	Trp^a^	BG2087 (*mtrB+*)	BG4233 (*ΔmtrB*)	Fold TRAP Regulation^c^	Fold tryptophan Regulation^d^
Wild Type (WT)	−	106±19	286±33	50±12	18±3
	+	6±2	312±56		
AU Top Stem	−	102±32	325±42	65±2	20±4
	+	5±0.5	344±57		
Mismatch Stem (MM)	−	119±22	260±33	19±3	9±1
	+	14±4	262±36		
Disrupted Stem (DS)	−	87±3	299±16	15±2	4±1
	+	20±4	293±12		
U-stretch Disruption (US)	−	245±18	444±47	11±2	7±0.4
	+	37±5	448±43		
GC Stretch	−	105±33	256±53	17±3	7±2
	+	15±0.5	299±47		

*^a^* − cells grown in the absence of exogenous L-tryptophan; +, cells grown in the presence of 50 µg/ml of L-tryptophan.

*^b^* Values are the average of two or three independent experiments, each performed in triplicate, ± the standard deviation.

*^c^* To determine fold regulation by TRAP, the ratio of BG4233 Trp- to BG2087 Trp+ was determined for each experiment, and the value shown is the average +/− the standard deviation.

*^d^* Fold tryptophan regulation was determined by taking the ratio of BG2087 Trp- to BG2087 Trp+ for each experiment, and the value shown is the average +/− the standard deviation.

Replacing the G116-C125 pair in the upper portion of the attenuator stem with an AU pair (Figure 2A AU Top Stem) slightly increased the apparent overall TRAP-mediated regulation of transcription from 50-fold to 65-fold (Table 1). However, the β-galactosidase values for the AU Top Stem mutant are all within the standard deviation of those for the WT fusion suggesting that TRAP-mediated termination was not significantly affected by this mutation. Disrupting the G116–C125 pair (Table 1 Mismatch Stem) resulted in two-fold less termination in the presence of TRAP and tryptophan. Nevertheless, TRAP induced 19-fold termination at this mutant attenuator. Further disrupting the stem with 6 mismatches (Figure 2A DS), had nearly the same modest effect as the single mismatch at G116–C125, showing substantial termination in the presence of TRAP and tryptophan (Table 1, Disrupted Stem).

The U-rich segment following the stem-loop of the *trp* attenuator is interrupted twice, at A136 and G140, which are the 4^th^ and 8^th^ residues respectively after the stem (Figure 2A). Our prior studies have shown that these interruptions also contribute to the weakness of the attenuator as an intrinsic terminator [12]. We made two substitutions in this portion of the attenuator (Figure 2A). Creating an XbaI site (TCTAGA) starting at residue 133 (Figure 2A U-stretch Disruption) generates a template in which three of the encoded U residues in the U-stretch at positions 134, 137 and 138 are substituted with C, G and A respectively (Figure 2A U-stretch Disruption). Similarly, creating a NotI site (GCGGCCGC) starting at residue 134 of the template creates a segment composed entirely of Gs and Cs for 8 residues after the stem (Figure 2A GC-stretch).

Both changes to the U-stretch resulted in decreased overall regulation, however significant TRAP-mediated transcription termination still occurred at both of these mutant attenuators (Table 1). The U-stretch Disruption displayed elevated transcription of *lacZ* in the absence of TRAP (BG4233 U-stretch Disruption: 444U), as compared to the WT attenuator (BG4233 WT: 286U). This observation suggests that a small amount of termination occurs at the WT attenuator in the absence of TRAP. In the presence of TRAP and exogenous tryptophan, TRAP induced termination at this mutant attenuator so as to regulate transcription 11-fold (Table 1 U-stretch Disruption). A more drastic change to the U-stretch, replacing it entirely with Gs and Cs, had relatively little effect as compared to the WT attenuator (Table 1 GC stretch). These results show that significant TRAP-mediated termination can take place in the absence of the U-stretch immediately downstream of the stem-loop *in vivo*. The location where termination occurs in *trp* leader regions containing altered attenuators will be addressed below.

### TRAP induces efficient transcription termination *in vivo* within the *trp* leader region in which the attenuator segment is deleted

**Figure 3:**
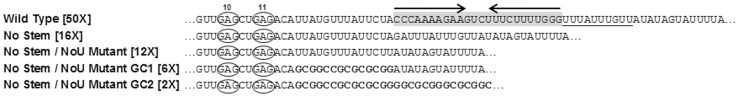
Diagram depicting deletion mutations in the stem-loop and U-stretch of the *trp* attenuator. The RNA segment that comprises the *trp* attenuator stem-loop is highlighted by a grey box with arrows indicating the complementary residues that form of the base-paired stem. Residues in the U-stretch of the attenuator are underlined. The sequences in bold show the G+C substitutions made in the regions downstream of the attenuator. The two most downstream of the 11 (G/U)AG triplet repeats (10 and 11) of the TRAP binding site are circled. The TRAP binding site was not altered by any of these deletion mutations. Numbers adjacent to structure names indicate fold TRAP regulation observed *in vivo* using β-galactosidase assays (results in Table 2).

The results in Table 1 show that TRAP is able to induce transcription termination in the *trp* leader region despite significant alterations to the stem-loop or to the U-rich tract of the attenuator. To further examine the requirement for *cis*-acting elements in TRAP-mediated transcription termination, various segments of the *trp* leader region were deleted and the ability of TRAP to induce termination *in vivo* was examined. Deleting residues +108 to +132 removes the entire stem-loop of the *trp* attenuator (Figure 3 No Stem). The only significant difference between the No Stem mutant and WT attenuator fusions was slightly less TRAP-mediated termination in the presence of tryptophan with the No Stem mutant (Table 2 BG2087: No Stem). Deleting residues +108 to +142 removes the entire *trp* attenuator including both the stem-loop and the U-rich tract (Figure 3 No Stem/No U). TRAP was only slightly less effective at inducing termination within this mutant leader region such that there was a 12-fold reduction in transcription continuing past the leader region into *lacZ* (Table 2 No Stem/No U, compare BG4233: 399U to BG2087+tryptophan: 33U).

**Table 2:** **Effects of deletions within the** ***trp*** **leader region on TRAP-mediated transcription termination** ***in vivo***.

		β-Galactosidase activity (U)^b^			
Regulatory Region	Trp^a^	BG2087 (*mtrB+*)	BG4233 (*ΔmtrB*)	Fold TRAP Regulation^c^	Fold tryptophan Regulation^d^
Wild Type (WT)	−	106±19	286±33	50±12	18±3
	+	6±2	312±56		
No Stem	−	104±2	264±40	16±0.4	7±1
	+	16±2	257±36		
No Stem/No U	−	128±17	399±145	12±1	4±1
	+	33±14	396±166		
No Stem/No U GC1	−	194±49	466±83	6±1	2±0.2
	+	82±27	477±87		
No Stem/No U GC2	−	202±14	391±22	2±0.1	1±0.1
	+	187±6	399±16		

*^a^* − cells grown in the absence of exogenous L-tryptophan; +, cells grown in the presence of 50 µg/ml of L-tryptophan.

*^b^* Values are the average of two or three independent experiments, each performed in triplicate, ± the standard deviation.

*^c^* To determine fold regulation by TRAP, the ratio of BG4233 Trp- to BG2087 Trp+ was determined for each experiment, and the value shown is the average +/− the standard deviation.

*^d^* Fold tryptophan regulation was determined by taking the ratio of BG2087 Trp- to BG2087 Trp+ for each experiment, and the value shown is the average +/− the standard deviation.

Examining the sequence of the No Stem/No U *trp* leader region revealed that deleting the entire attenuator results in the presence of an A+U-rich segment of nearly 30 residues immediately downstream of the TRAP binding site (Figure 3 No Stem/No U). Hence, if the U-rich tract of the attenuator plays a role in TRAP-mediated termination, such as inducing RNAP to pause or weakening the DNA/RNA hybrid in the transcription elongation complex, these effects may still occur in the No Stem/No U mutant. To test this possibility, we created two additional templates based on the No Stem/No U mutant lacking the *trp* attenuator. In the first template (No Stem/No U GC1) the A+U-rich segment immediately following the TRAP binding site was replaced with a 14 base segment composed of Gs and Cs. In the second template (No Stem/No U GC2), the entire A+U-rich region after the TRAP binding site was replaced with a 28 residue GC segment. Replacing 14 residues of the A+U-rich segment proximal to the TRAP binding with a GC segment substantially reduced the ability of TRAP to induce termination in the leader region (Table 2 No Stem/No U GC1: 82U). Nevertheless, the presence of tryptophan-activated TRAP reduced transcription into *lacZ* by approximately 6-fold (Table 2). Replacing the 28 residues of the A+U-rich segment following the TRAP binding site with a segment composed of Gs and Cs nearly eliminated the ability of TRAP to affect termination in the *trp* leader region (Table 2 No Stem/No U GC2).

### Mapping the location of TRAP-mediated transcription termination within the mutant *trp* leader regions

The results presented in Tables 1 and 2 show that TRAP induces significant transcription termination prior to *lacZ* in nearly all the *trpE′-′lacZ* fusions we tested, even those that contain drastic alterations to the attenuator. However, these studies do not provide information about the location within the *trp* sequences where transcription terminates. If the attenuator is responsible for directing where TRAP mediates termination, then making substitutions within the attenuator region may alter this location. Hence, we used RNase protection assays to determine where TRAP-mediated transcription termination is occurring in these mutant leader regions.

**Figure 4:**
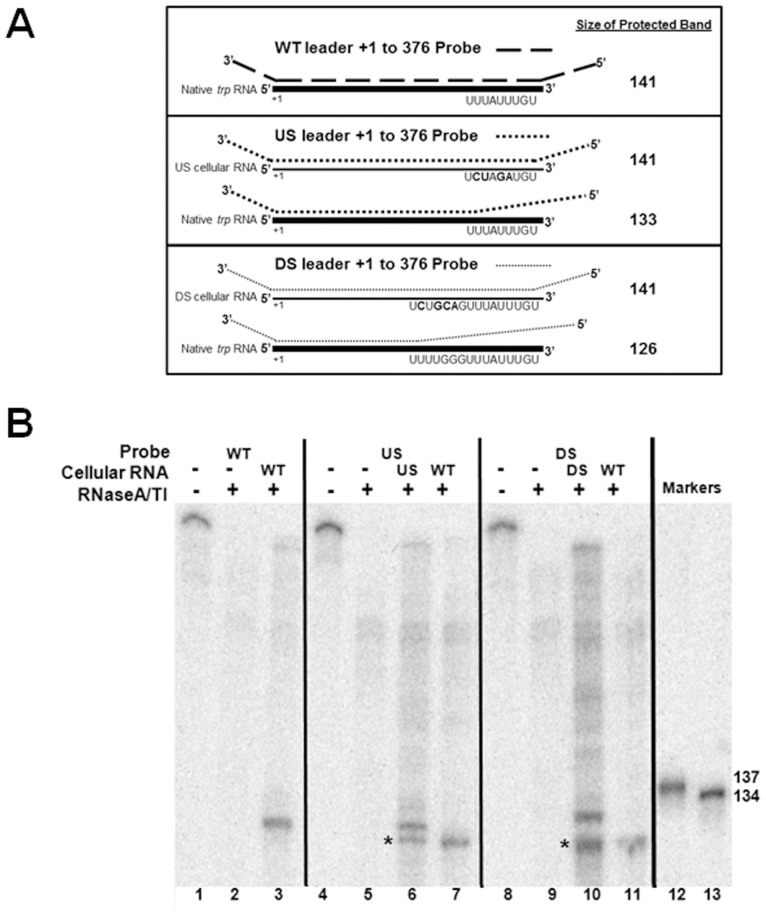
Mapping the site of TRAP-mediated transcription termination *in vivo*. **(A)** Schematic diagram of RNase protection assays (RPA) using ^32^P-labeled antisense probes and cellular RNA from *B. subtilis*. Antisense RNA probes are indicated as dotted lines and cellular RNA is displayed as solid lines (thick line for WT *trp* RNA and thin line for RNA from the mutant *trpE′-′lacZ* fusions). The WT antisense probe was hybridized to WT cellular RNA, and the mutant antisense probes, U-stretch (US) mutant or Disrupted stem (DS), were hybridized to RNA from *B. subtilis* containing mutant *trpE′-′lacZ* fusions, as well as to RNA from WT cells. The predicted lengths of the protected products are shown at the right side of the figure. **(B)** RNase protection assays to determine location of transcription termination in *trp* leader regions containing mutant attenuators. WT and mutant *trp* antisense RNA (Probes) were incubated with cellular RNA from WT *B.subtilis* or strains containing *trpE′-′lacZ* fusions with either the U-stretch (US) mutant or Disrupted stem (DS) mutant attenuator prior to digestion with RNaseT1/A. Black arrow represents the location of transcripts terminated at the WT attenuator; (*) indicates bands that are from probes designed to hybridize with transcripts terminated at mutant attenuator present in *trpE′-′lacZ* fusion when they pair with native WT *trp* leader transcripts terminated at the WT attenuator. Reactions were run on 8% denaturing polyacrylamide gels. Markers were generated by T7 RNAP run off transcription of templates that contain an XbaI site from +133–138, or a PstI site from +134–139 relative to the start of transcription respectively. In each case a T7 promoter was placed upstream of the template so that transcription initiation occurs at +1. The 137 nt RNA marker was generated by transcription of the XbaI cleaved template, and the 134 nt RNA marker was from template cut with PstI.

When WT *B. subtilis* is grown in the presence of tryptophan, the majority of transcripts originating at the *trp* promoter terminate at the attenuator within the leader region [10]. Since the goal of our RNase protection assays was to determine whether TRAP induces termination at the same location in WT and several mutant *trp* leader regions, we only examined *trp* transcripts produced from cells grown in excess tryptophan. Studies by Yakhnin and Babitzke have established that *in vitro* attenuated *trp* transcripts terminate at U141 and G140 in the absence and presence of NusA respectively [5]. Hence, our antisense probe, which is complementary to from +1 to +376 is expected to be protected from +1 to approximately +141 [5] (Figure 4A). The full length WT antisense probe in the absence of RNase is seen in lane 1 (Figure 4B lane 1). RNase treatment in the absence of cellular RNA resulted in complete degradation of the labelled probe (Figure 4B lane 2). As expected, RNase treatment after hybridizing the WT *trp* leader probe with cellular RNA from WT cells grown in excess tryptophan yielded a single protected band of approximately the expected size for transcripts ending at the attenuator (Figure 4B lane 3).

The *B. subtilis* strains harbouring *trpE′-′lacZ* transcriptional fusions also contain transcripts from the native WT *trp* operon and hence produce two different types of transcripts containing the *trp* leader region. For each mutant, an antisense probe complementary to the mutant *trp* attenuator contains several mismatches with the 3′ region of RNAs terminated at the WT attenuator (Figure 4A). This situation provides an internal control to compare the location where termination occurs within the leader regions with altered attenuator sequences to the site of termination with the WT attenuator.

The U-stretch Disruption (US) probe is complementary to +1 to +141 of transcripts from the US mutant leader region from the *trpE′-′lacZ* fusion (Figure 4A). If TRAP induces transcription termination at a similar location in this mutant leader region as it does in the presence of the WT attenuator, then the US probe should yield a protected fragment of similar size to that seen with the WT probe with WT RNA (Figure 4A). When this probe hybridizes to native *trp* transcripts terminated at the WT attenuator, the 3′-most 8 residues of the transcript do not pair with the US probe. Hence the protected fragment corresponding to the native *trp* transcript is predicted to be 8 residues shorter (Figure 4A). When the US probe was hybridized with cellular RNA from the strain with the U-stretch (US) mutant *trpE′-′lacZ* fusion, two protected bands are seen (Figure 4B compare lane 3 and lane 6). One of these bands is very similar in length as that seen with the WT probe hybridized to WT cellular RNA (Figure 4B compare land 6 and lane 3). The second band, labelled with a single asterisk, is slightly smaller, as predicted if the US mutant probe hybridizes to the native *trp* transcript (Figure 4A). The identity of the lower band as resulting from protection by native *trp* operon transcripts was confirmed by hybridizing the US mutant probe with RNA isolated from WT *B. subtilis* lacking a *trpE′-′lacZ* fusion. A single protected band that co-migrates with the lower band in lane 6 was observed (Figure 4B lane 7).

Similar results were obtained with a probe designed to hybridize to *trp* mRNAs with the Disrupted Stem (DS) mutant attenuator (Figure 4B). When this probe was hybridized to RNA from the *B. subtilis* strain harbouring the DS *trpE′-′lacZ* fusion, two protected bands are observed (Figure 4B lane 10). The top band corresponds to fully protected transcripts from the DS *trpE′-′lacZ* fusion. The identity of the lower band as resulting from hybridization with native *trp* transcripts was again confirmed using RNA from WT *B. subtilis* (Fig. 4B lane 11). Together these results demonstrate that transcripts from the *trpE′-′lacZ* fusion containing the U-stretch (US) or the Disrupted stem (DS) mutant attenuator terminate at approximately the same location as transcripts that terminate at the WT attenuator.

In lanes 6 and 10 strong bands are seen corresponding to transcripts terminating at the US and DS mutant attenuators whereas little or no fully protected probes resulting from transcripts that read through the leader region into *trpE* are observed. These observations support the conclusion that the changes in β-galactosidase activity observed when cells containing these fusions are grown in the presence of tryptophan reflect increased transcription termination in the leader region.

### TRAP does not cause efficient transcription termination within the mutant *trp* leader regions *in vitro*

**Figure 5:**
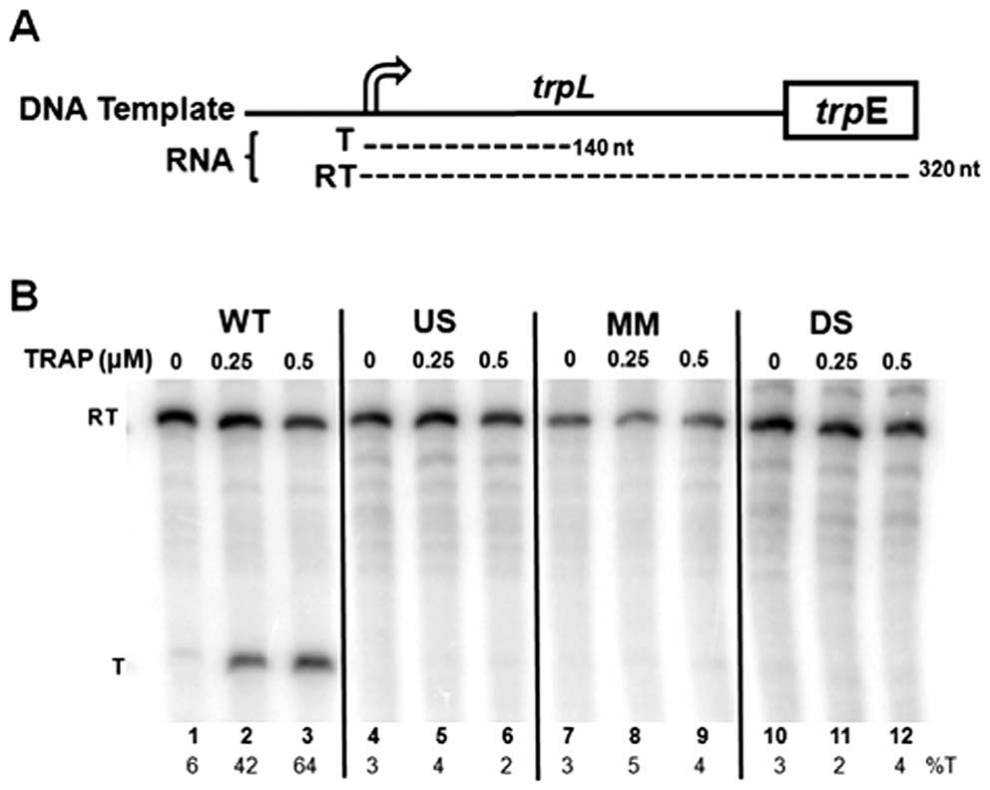
*In vitro* transcription attenuation assays. **(A)** Diagram of DNA templates used for *in vitro* transcription attenuation assays. The two major transcripts produced from these templates include read through (RT; ∼140 nts) and terminated (T: ∼320 nts) transcripts are shown below the diagram of the template. **(B)** 6% polyacrylamide-8M urea gel electrophoresis analysis of the products of *in vitro* transcription of the wild type (WT), U-stretch (US), mismatch stem (MM), and disrupted stem (DS) templates in the absence and presence of 0.25 or 0.5 µM TRAP. Positions of read through (RT = 320 nt) and terminated (T = 140 nt) transcripts are indicated at left side of the figure. The percentage of transcripts terminating at the attenuator (%T) for each reaction is shown at the bottom of each lane.

The ability of TRAP to induce transcription termination at three mutant *trp* attenuators was also examined *in vitro* using an attenuation assay described previously [12]. The DNA template used for these assays contained the *trp* promoter and leader region, including the WT or mutant attenuator, as well as the first 45 codons of the *trpE* gene (Figure 5A). Transcription of the WT template with *B. subtilis* RNAP yielded two major RNAs, corresponding to transcripts that terminate at the attenuator (T:140 nt), as well as those that read through the attenuator and continue to the end of the template (RT:320 nt) (Figure 5B). In the absence of TRAP, mostly read through transcripts were produced with only 6% terminated at the attenuator (Figure 5B lane 1). Addition of increasing amounts of tryptophan-activated TRAP up to 0.5 µM increased the fraction of transcripts that terminated at the attenuator to 64% (Figure 5B lane 3). These observations are similar to those seen previously [5], [12] and are indicative of TRAP-mediated termination at the *trp* attenuator.

In contrast to the *in vivo* results (Tables 1 and 2), TRAP did not induce significant termination at any of the three mutant attenuators tested in this minimal *in vitro* transcription system (Figure 5 lanes 4–12). These include attenuators with alterations in either the U-stretch (Figure 5 US: lanes 4–6) or in the base-paired stem (Figure 5 MM & SD: lanes 7–12). These observations suggest that there is something missing from or different in this *in vitro* attenuation assay that allows TRAP to induce termination at these mutant attenuators *in vivo*.

### NusA and the timing of TRAP binding to the nascent RNA are important in TRAP-mediated transcription termination

**Figure 6:**
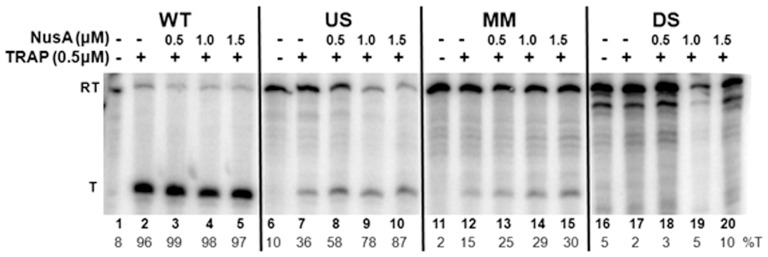
Effects of reduced NTP levels and of NusA on TRAP-mediated transcription termination *in vitro*. Polyacrylamide gel electrophoresis analysis of *in vitro* transcription of templates with the Wild Type (WT), U-stretch (US), mismatch stem (MM), and disrupted stem (DS) *trp* attenuator regions. Transcription reactions were performed in the absence or presence of 0.5 µM TRAP, and 0–1.5 µM NusA. The positions of read through (RT) and terminated (T) transcripts are indicated at the left. The percentage termination (%T) for each reaction is at the bottom of each lane.

The timing of TRAP binding to the nascent *trp* transcript relative to the position of the transcribing RNAP is crucial for attenuation [24]. TRAP must bind to the *trp* mRNA before RNAP transcribes past the attenuator region if transcription termination is to occur. Hence, we examined whether the inability of TRAP to induce termination at the mutant *trp* attenuators *in vitro* is because RNAP has progressed too far before TRAP binds to these nascent transcripts. The first approach was to slow the rate of transcription elongation in the *in vitro* attenuation assay by reducing the concentration of NTPs [25], [26]. Reducing the NTP concentration from 500 µM to 50 µM increased transcription termination in the presence of 0.5 µM TRAP at the WT attenuator from 64% (Figure 5 lane 3) to 96% (Figure 6 lane 2). At the lower NTP concentration, TRAP-mediated termination also increased at the mutant attenuator with alterations in the U-stretch (compare Figure 6 lane 7 with Figure 5 lane 7). In addition, there was a slight increase in TRAP-mediated termination to 15% with the attenuator containing a single mismatch at the top of the stem (Figure 6 lane 12). However, lowering the NTP concentration did not yield substantial TRAP-mediated termination at the attenuator with the disrupted stem *in vitro* (Figure 6 lanes 17).

Previous studies have shown that NusA stimulates RNAP pausing at positions 107 and 144 within the *B. subtilis trp* leader region [4], [22], [27]. Pausing at position 107 has been suggested to provide time for TRAP to bind the nascent RNA before RNAP progresses beyond the attenuator region [22], [27]. Hence, we tested whether the presence of NusA could enhance TRAP-mediated termination at these mutant attenuators. NusA significantly increased TRAP-mediated transcription termination at the attenuator with changes in the U-stretch (Figure 6 lanes 8–10), such that at the highest level of NusA nearly all (87%) of the transcripts produced end at the attenuator. A two-fold increase in TRAP-mediated termination was also observed in the presence of NusA at the attenuator with a single mismatch in the stem yielding at most 30% termination (Figure 6 lanes 12–15). However, NusA showed no effect on TRAP-mediated termination at the mutant attenuator with the disrupted stem (Figure 6 lanes18–20).

Finally, to ensure sufficient time for TRAP to bind the nascent transcript before RNAP progresses beyond the attenuator, we blocked the transcription elongation complex (TEC) with EcoRI* bound to the DNA [21]. When the cleavage defective E111Q EcoRI protein (EcoRI*) is bound to the DNA template, it blocks the TEC such that the nascent transcript up to approximately 26–27 nucleotides upstream of the first G in the EcoRI site is exposed on the nascent transcript [28]. Blocked TECs containing *B. subtilis* RNAP remain stably bound to the DNA template and competent to resume transcription for at least 1 hour (S. Sharma & P. Gollnick unpublished observations). Adding 0.5 M KCl dissociates EcoRI* from the DNA, releasing the blocked TECs to continue transcribing the template DNA.

**Figure 7:**
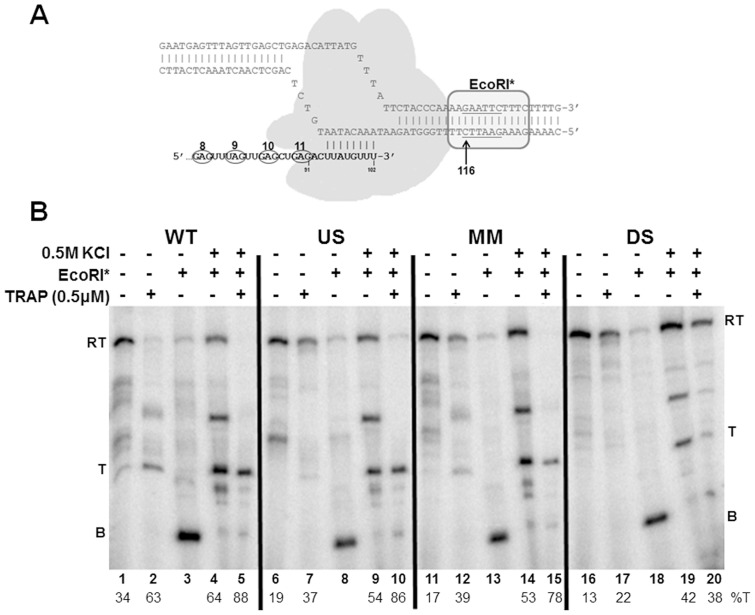
Blocking transcription elongation with EcoRI* to provide time for TRAP binding. **(A)** Diagram of EcoRI* blocking the transcription elongation complex. Cleavage defective E111Q EcoRI (EcoRI*) binds to its recognition site on the DNA template and blocks elongation of RNAP approximately 12–13 bp upstream of the first G of the GAATTC recognition site. EcoRI* is shown as an oval shape bound to its recognition site starting at +116 in the *trp* leader region. RNAP is shown as a shaded grey shape. The nascent RNA is shown in bold with the 3′-most 8 residues paired with the template DNA. The last 4 (G/U)AG repeats of the TRAP binding site are circled. **(B)** Gel electrophoresis using a 6% polyacrylamide-8M urea gel analysis of block-and-release assay for the wild type (WT), U-stretch Disruption (US), C125G mismatch stem (MM), and disrupted stem (DS) attenuator templates. EcoRI* was allowed to bind to the DNA template prior to initiating transcription, TRAP was added, and transcription was then allowed to proceed until the TECs were blocked by EcoRI*. EcoRI* was then dissociated from the DNA by addition of 0.5 M KCl, allowing transcription to resume. The location of transcripts from blocked TECs (B), terminated at the attenuator (T), and read through (RT) transcripts are indicated on both sides of the gel. The percentage of transcription termination is displayed below the lane numbers (%T).

For these studies, we constructed a template for *in vitro* transcription assays that is identical to that described in Figure 5A but with an EcoRI site (GAATTC) starting at +116 (Eco116) within the *trp* leader region [12]. When EcoRI* is bound to this template, it blocks RNAP before it reaches the attenuator region such that nearly the entire TRAP binding site is exposed on the nascent transcript (Figure 7A). Hence, when TRAP is added to blocked TECs on this template it can stably bind to the 10 exposed (G/U)AG repeats in the nascent RNA (S. Sharma and P. Gollnick submitted). Dissociating EcoRI* with KCl then allows transcription to resume with TRAP bound to the nascent RNA. This approach ensures that TRAP is bound to the transcript before the transcribing RNAP progresses beyond the attenuator region.

Transcription of the Eco116 template containing the WT *trp* attenuator yields two major transcripts that are similar to those seen previously with the WT template (see Figures 2 and 5). These RNAs correspond to transcripts that terminate at the attenuator (Figure 7B: T), and those that read through the attenuator and continue to the end of the template (Figure 7B: RT). In addition a number of other bands corresponding transcripts halted after the attenuator and before the end of the template are seen that were not seen in Fig. 6. These may be due to the increased KCl used to dissociate EcoRI*. In the absence of TRAP, the majority of the transcripts (66%) produced read through the WT attenuator (Figure 7B lane 1), whereas in the presence of TRAP most (63%) of the transcripts halted at the attenuator (Figure 7B lane 2). These observations are similar to those seen in Figure 5 and indicate that the presence of the EcoRI site in this template did not significantly alter TRAP-mediated regulation of transcription. In the presence of EcoRI*, nearly all the TECs are blocked at +116 yielding a 104 nt transcript (Figure 7B lane 3 labelled B). Removing the EcoRI* roadblock with 0.5 M KCl allows transcription to resume. Doing so in the absence of TRAP resulted in 64% of transcriptions terminating at the attenuator (Figure 7B lane 4). Resuming transcription in the presence of TRAP resulted in 88% of the TECs halting at the attenuator (Figure 7B lane 5). Although increased termination at the attenuator in the absence of TRAP was observed using this block-and-release approach (lane 4) as compared to transcription of the same template in the absence of the block (lane 2), increased TRAP-mediated termination is apparent in lane 5 (Figure 7).

Using this block-and-release assay also resulted in increased TRAP-mediated termination at the mutant attenuator with changes in the U-stretch (US Figure 7 lanes 6–10), and the attenuator with a single mismatch in the base-paired stem (MM Figure 7 lanes 11–15). However, TRAP was not able to induce significant termination above that seen upon resuming transcription in the absence of TRAP at the attenuator with the disrupted stem (DS Figure 7 lanes 16–20).

Together, the results in Figures 5, 6, and 7 show that the inability of TRAP to induce termination *in vitro* within the U-stretch (US) mutant, or within the attenuator containing a single mismatch in the stem (MM) is, at least in part, related to the timing of TRAP binding relative to the position of the transcribing RNAP. However, none of the approaches used to provide additional time for TRAP to bind to the nascent transcript before RNAP has extended beyond the attenuator region, resulted in increased termination attenuator with the disrupted stem (DS). In contrast, TRAP induced termination at this DS attenuator *in vivo* such that transcription of *lacZ* was reduced 15 fold (Table 1 Disrupted Stem). Together these observations suggest something may be missing from our *in vitro* system that allows TRAP to function at this mutant attenuator in vivo.

## Discussion

In this study, we have further examined the role of the *trp* attenuator RNA in TRAP-mediated transcription termination. Our results show that TRAP can induce efficient termination within the *trp* leader region *in vivo* when the number of U residues in the U-stretch is reduced or if the hairpin stem is disrupted (Table 1). Substantial TRAP-mediated termination even occurred *in vivo* when the *trp* attenuator was deleted entirely (Table 2). The only clear requirement for TRAP to induce termination *in vivo* is an A+U-rich region immediately downstream of the TRAP binding site (Table 2). This requirement suggests that RNAP pausing in this region may play a role in TRAP-mediated termination. Pausing of RNAP is thought to be the first step in intrinsic termination pathway, and is a prerequisite for efficient termination [29]–[32]. Pausing is primarily induced by the U-stretch and allows formation of the terminator hairpin [33]. Similarly, pausing may facilitate the interaction of TRAP with RNAP. Moreover, this pausing would take place while the A+U-rich RNA is within the RNA/DNA hybrid, which may facilitate shearing the DNA-RNA hybrid when TRAP induces forward translocation of the paused RNAP [12].

Prior studies from the Yanofsky lab using similar *trpE′-′lacZ* fusions also found evidence that TRAP can regulate transcription through the *trp* leader region in which the attenuator (terminator) is deleted [11]. For these studies in additional to examining β-galactosidase activity, *lacZ* mRNA levels were measured directly to assess transcriptional regulation independent of changes in translation. They found that TRAP mediated approximately 9-fold reduction in mRNA levels from a similar fusion in which the *trp* attenuator (+108 to +133; See Figure 1) was deleted [11]. This level is quite similar to the 12-fold regulation that we observed for the No Stem/No U fusion (Table 2), which contains a nearly identical (+108 to +132) deletion in the leader region. Merino *et al.* stated they had no clear explanation for this residual 9-fold TRAP-mediated decrease in mRNA levels in the absence of the attenuator [11]. However, they speculated that this strain retains some TRAP-dependent transcription termination *in vivo*. Our results clearly support this proposal.

In contrast to our observations concerning the role of the attenuator in TRAP-mediated termination *in vivo*, even minor changes to the attenuator stem (MM) or U-stretch (US) abolished TRAP-mediated termination in a simple *in vitro* transcription attenuation assay using *B. subtilis* RNAP (Figure 5B). Several modifications of the *in vitro* transcription system to provide additional time for TRAP to bind the nascent RNA before RNAP has extended beyond the attenuator region increased TRAP-mediated termination at an attenuator with changes in the U-stretch (Figures 6 and 7). These observations are consistent with the role of the U-stretch being to induce RNAP to pause as the first step in intrinsic termination [33]. Hence, if pausing is a requisite feature of TRAP-mediated termination as well, then deficiencies in U-stretch induced pausing could be ameliorated by other means to provide adequate time for TRAP to bind. The observation that addition of NusA restores TRAP-mediated termination with the US mutant is also consistent with NusA enhancing transcriptional pausing [22], [27].

None of our efforts to enhance the *in vitro* attenuation assay allowed TRAP to induce significant termination at the disrupted stem (DS) attenuator. In contrast, TRAP induces significant termination at the DS attenuator *in vivo* (Table 1). One explanation for this difference could be that another protein factor is required for termination at this attenuator, and this factor is missing in our *in vitro* system. We attempted a genetic search using transposon mutagenesis for additional genes whose disruption reduces TRAP-mediated termination. However, thus far we have not identified any such genes. It is possible that this factor is essential and thus disruption of its gene is lethal.

**Figure 8:**
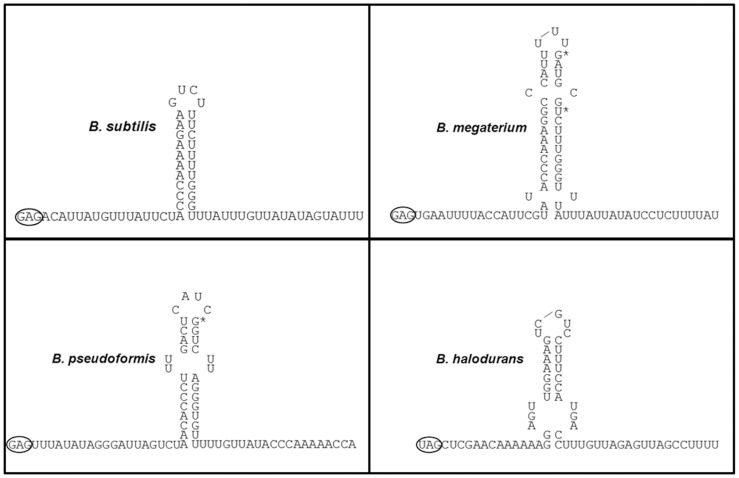
*Trp* attenuator regions of different bacterial species. Predicted attenuator RNA structures from the *trp* leader region of several TRAP containing bacterial species using M-fold [46]. The last repeat in the proposed TRAP binding is circled and the predicted attenuator structure is shown as well as 20 residues downstream of the predicted base-paired stem.

In addition to finding that the attenuator is not essential for TRAP-mediated transcription termination *in vivo*, efforts to demonstrate that the predicted attenuator structure forms in *trp* leader region RNA have been unsuccessful. Attempts to map the RNA structure of the *trp* attenuator, either in the presence of TRAP bound to the leader region RNA or by using mutant leader regions in which the antiterminator is disrupted or deleted, have only provided evidence for base-pairing of the three GC base pairs at the base of the stem (N. Merlino and P. Gollnick unpublished observations). In contrast, the other predicted RNA structures in the *trp* leader region, including the 5′ stem-loop, the antiterminator and the ribosome binding site sequestering structure, have been extensively mapped [34]–[36]. This inability to structure map the attenuator hairpin is surprising given the predicted ΔG of −12.6 kcal/mol for formation of this structure [37], but is consistent with the weak intrinsic termination activity of the attenuator. The *trp* leader regions from several other species of bacteria that contain a gene encoding TRAP and contain a predicted TRAP binding site upstream of *trpE*, also contain predicted attenuators that appear unlikely to function as intrinsic terminators. These attenuators also display low GC content in their base-paired stems and interruptions in their U-stretches. Moreover, three species including *B. megaterium, B. pseudoformis*, and *B. halodurans*, have predicted attenuators with multiple mismatches in their predicted hairpin stems (Figure 8). In particular the predicted *B. halodurans trp* attenuator has a weak stem-loop structure (ΔG = −8.4 kcal/mol), and contains three mismatches near the base of the predicted stem [23]. Nevertheless, transcription has been shown to terminate at or near this site in the presence of TRAP and tryptophan [23]. These observations suggest that these attenuators, like that of the *B. subtilis trp* operon, likely play a minor role in TRAP-mediated control of transcription. The leader regions from these bacilli also contain A+U-rich segments both upstream and downstream of the predicted attenuator similar to that in the *B. subitlis trp* leader, which we have shown is important for TRAP-mediated termination. Hence, it seems likely that the mechanism of TRAP-mediated attenuation in these species is similarly to that we have described for *B. subtilis*.

In the forward translocation model of intrinsic termination, the U-stretch following the hairpin stem acts to pause RNAP, and formation of the hairpin stem induces RNAP to move forward along the DNA [29]. This forward movement displaces the RNA from the active site, shears the DNA-RNA hybrid, and transcription terminates [30]. If the hairpin stem is weak due to low GC content and if the U-stretch is interrupted, as in the case of the *trp* attenuator, forward translocation may not occur efficiently [30]. Thus TRAP binding to the nascent RNA may enhance forward movement of the RNAP to cause termination [12]. This model is similar to the proposed function of other bacterial termination factors such as Mfd and Rho [38]. In addition, we have shown *in vivo* that TRAP-mediated transcription termination within the leader region does not require the attenuator, but does require an A+U-rich region following the TRAP binding site, which may serve to pause RNAP. Our revised model for transcription termination within the *trp* leader region suggests that TRAP functions not only to alter the RNA secondary structure but also to directly induce RNAP to terminate transcription, possibly by directly interacting with RNAP. Consistent with this model, we have recently isolated a TRAP (E60K) mutant that binds tryptophan and RNA with similar properties as the WT protein but shows 3-fold less termination in the *trp* leader region in cells grown in the presence of excess tryptophan. Hence Glu60 may interact with RNA polymerase to induce termination.

As recently pointed out by Peters *et al*., the majority of mechanistic studies of intrinsic transcription termination are based on a small set of model terminators mostly from *E. coli* and closely related bacteria [39]. Termination mechanisms likely differ in other bacteria since the genomes of some species including *Mycobacterium, Helicobacter, Treponema, Synechocystis, Mycoplasma*, and *Borrelia* do not appear to contain canonical terminator structures downstream of their genes [40], [41]. Moreover, alternative types of predicted RNA structures have been identified downstream of coding regions [42]. These structures may function in as of yet uncharacterized termination mechanisms, which could involve termination proteins [43]. Conversely, there are other examples of RNA structures that resemble canonical intrinsic terminators yet fail to function as such. In addition to the *B. subtilis trp* attenuator, the *E. coli trpA* and *asnU* genes contain features that resemble intrinsic terminators at the end of these genes but do not induce termination. The *E. coli asnU* gene is followed by a GC rich hairpin stem and a U-stretch, however termination of transcription of this gene is Rho-mediated [44]. The *E. coli trp* operon contains an apparent intrinsic terminator after *trpA*. However, termination is inefficient at this site and occurs further downstream by Rho-dependent termination [45]. Hence, the TRAP-mediated termination may represent a new type of factor dependent termination mechanism that will be found to be common in other bacterial species.
